# Disparities in Coronavirus 2019 Reported Incidence, Knowledge, and Behavior Among US Adults

**DOI:** 10.1001/jamanetworkopen.2020.12403

**Published:** 2020-06-18

**Authors:** Marcella Alsan, Stefanie Stantcheva, David Yang, David Cutler

**Affiliations:** 1 Harvard Kennedy School, Cambridge, Massachusetts; 2 The Bureau for Research and Economic Analysis of Development, Cambridge, Massachusetts; 3 The National Bureau of Economic Research, Cambridge, Massachusetts; 4 Department of Economics, Harvard University, Cambridge, Massachusetts

## Abstract

**Importance:**

Data from the coronavirus disease 2019 (COVID-19) pandemic in the US show large differences in hospitalizations and mortality across race and geography. However, there are limited data on health information, beliefs, and behaviors that might indicate different exposure to risk.

**Objective:**

To determine the association of sociodemographic characteristics with reported incidence, knowledge, and behavior regarding COVID-19 among US adults.

**Design, Setting, and Participants:**

A US national survey study was conducted from March 29 to April 13, 2020, to measure differences in knowledge, beliefs, and behavior about COVID-19. The survey oversampled COVID-19 hotspot areas. The survey was conducted electronically. The criteria for inclusion were age 18 years or older and residence in the US. Data analysis was performed in April 2020.

**Main Outcomes and Measures:**

The main outcomes were incidence, knowledge, and behaviors related to COVID-19 as measured by survey response.

**Results:**

The survey included 5198 individuals (mean [SD] age, 48 [18] years; 2336 men [45%]; 3759 white [72%], 830 [16%] African American, and 609 [12%] Hispanic). The largest differences in COVID-19–related knowledge and behaviors were associated with race/ethnicity, sex, and age, with African American participants, men, and people younger than 55 years showing less knowledge than other groups. African American respondents were 3.5 percentage points (95% CI, 1.5 to 5.5 percentage points; *P* = .001) more likely than white respondents to report being infected with COVID-19, as were men compared with women (3.2 percentage points; 95% CI, 2.0 to 4.4 percentage points; *P* < .001). Knowing someone who tested positive for COVID-19 was more common among African American respondents (7.2 percentage points; 95% CI, 3.4 to 10.9 percentage points; *P* < .001), people younger than 30 years (11.6 percentage points; 95% CI, 7.5 to 15.7 percentage points; *P* < .001), and people with higher incomes (coefficient on earning ≥$100 000, 12.3 percentage points; 95% CI, 8.7 to 15.8 percentage points; *P* < .001). Knowledge of potential fomite spread was lower among African American respondents (−9.4 percentage points; 95% CI, −13.1 to −5.7 percentage points; *P* < .001), Hispanic respondents (−4.8 percentage points; 95% CI, −8.9 to −0.77 percentage points; *P* = .02), and people younger than 30 years (−10.3 percentage points; 95% CI, −14.1 to −6.5 percentage points; *P* < .001). Similar gaps were found with respect to knowledge of COVID-19 symptoms and preventive behaviors.

**Conclusions and Relevance:**

In this survey study of US adults, there were gaps in reported incidence of COVID-19 and knowledge regarding its spread and symptoms and social distancing behavior. More effort is needed to increase accurate information and encourage appropriate behaviors among minority communities, men, and younger people.

## Key Points

**Question:**

How do reported incidence, knowledge, and behaviors regarding coronavirus disease 2019 vary across sociodemographic characteristics in the US?

**Findings:**

In this survey study, the largest differences in coronavirus disease 2019–related knowledge and behaviors were associated with race/ethnicity, sex, and age. African American participants, men, and people younger than 55 years were less likely to know how the disease is spread, were less likely to know the symptoms of coronavirus disease 2019, washed their hands less frequently, and left the home more often.

**Meaning:**

These findings suggest that more effort is needed to increase accurate information and encourage appropriate behaviors among minority communities, men, and younger people.

## Teaser

This survey study examines the association of sociodemographic characteristics with reported incidence, knowledge, and behavior regarding coronavirus disease 2019 among US adults.

## Introduction

Rates of coronavirus disease 2019 (COVID-19) differ greatly across the population. Data from the US Centers for Disease Control and Prevention on COVID-19 hospitalization and mortality rates show a higher incidence among racial and ethnic minorities, male individuals, the elderly, and people living in less dense areas.^1^ These data are consistent with media reports^2,3,4,5,6,7^ from specific areas of higher hospitalization and death rates from COVID-19 among African American patients and individuals with lower socioeconomic status.

Public health efforts to reduce the spread of COVID-19 will thus need to focus particularly on these economically disadvantaged population groups. However, historically, such efforts have had difficulty making inroads into socially marginalized groups. For example, rates of smoking and obesity are higher among lower socioeconomic status groups, and trust in health care is lower among racial minorities.^8,9,10^ Furthermore, several polls have demonstrated a partisan divide whereby right-leaning media outlets and individuals who consume them are skeptical about the threat posed by COVID-19 and efforts to reduce its spread.^11,12^ This raises concerns that the response to COVID-19 will be hampered by differences in knowledge, beliefs, and behaviors among racial/ethnic minorities, groups with low socioeconomic status, or groups with differing political orientations in the population.

We conducted a large-scale survey in the US in late March and early April 2020, during the early exponential growth phase of the epidemic. The survey is nationally representative, with an oversampling of COVID-19 hotspot areas at the time. The survey asked questions about reported prevalence of COVID-19, knowledge of COVID-19, beliefs about the coronavirus, and behaviors associated with the spread of COVID-19. Results were used to determine how these measures differ by race/ethnicity, socioeconomic status, and political orientation.

## Methods

This survey study was reviewed by the institutional review board of Harvard University and was deemed exempt. All participants provided written, informed consent.

### Data

We developed and conducted a survey on the effects of COVID-19. Given the urgency to deploy the instrument, the survey was not extensively piloted before launch. The survey was conducted in the US from March 29 to April 13, 2020, and was performed online by Dynata Corporation. Refusal rates per American Association for Public Opinion Research reporting guidance cannot be calculated because the survey company uses its proprietary panel to recruit participants, and the research team only receives information on those who completed the questionnaire. Eighty percent of respondents were sampled from geographic areas according to population representation, and 20% were sampled from hotspot areas (ie, those with the highest counts of COVID-19 at the time): Seattle, Washington; New York, New York; New Orleans, Louisiana; and Detroit, Michigan. Exact question wording is shown in the eAppendix in the Supplement. Summary statistics on the sample are reported in eTable 1 in the Supplement. The survey was conducted by internet. The survey sample is very similar to the overall US population, with the exception of a higher share of individuals with a college degree in the survey than in the nation. Race/ethnicity was self-reported by the respondent. These data were collected to assess differences across various sociodemographic characteristics.

#### Questions About COVID-19 Interactions, Knowledge, and Behaviors

Two measures of direct interactions with COVID-19 were asked: whether the respondent had already contracted COVID-19, and whether the respondent personally knew someone who has contracted COVID-19. A number of questions were asked about knowledge of COVID-19. One question asked whether a person could infect others without being sick or showing symptoms. A separate question asked respondents to answer yes or no to 5 questions about COVID-19 spread: whether the virus spreads through close contact with an infected person (within approximately 6 feet), through respiratory droplets produced when an infected person coughs or sneezes, by touching a contaminated surface and then touching your eyes nose or mouth, through unprotected sex, and whether the virus is a hoax. Analysis focused particularly on knowledge of fomite transmission (a binary variable for whether the respondent knows that touching a contaminated surface can lead to transmission).

Respondents were also asked to identify the top 3 symptoms of COVID-19 from a list: fever, dry eyes, skin rash, cough, difficulty breathing, swollen legs, acid reflux, stomachache, and watery eyes. From these responses, a binary variable was created for whether the respondent knew all 3 COVID-19 symptoms (fever, cough, and difficulty in breathing). Behaviors to mitigate the spread of COVID-19 were measured by 2 variables: how many times the person washed their hands in the past 24 hours, and how many times the person left their house in the past 3 days.

#### Covariates

We examined the reported infection, knowledge of spread and symptoms, and behaviors associated with socioeconomic and political orientation variables. Demographic variables included age (included in regression models as <30, 30-54, 55-64, and ≥65 years), sex (male or female), race/ethnicity (non-Hispanic white, non-Hispanic black, or Hispanic), annual household income (included in regression models as <$25 000, $25 000-$49 999, $50 000-74,999, $75 000-$99 999, and ≥$100 000), and political orientation (Democratic, Republican, and independent or no affiliation). A dummy variable is also included for whether the individual lived in a hotspot area (New York City, Seattle, New Orleans, and Detroit).

### Statistical Analysis

Linear regression analysis was used to measure the association of reported incidence, knowledge, and behaviors with the aforementioned independent variables (eTable 2 in the Supplement). Additional controls included whether the individual had health insurance, whether the respondent reported a condition that placed them at higher risk of death if infected (eg, cardiovascular disease, chronic lung disease, or diabetes), a measure of risk tolerance (a 0-10 measure of willingness to take risk), exact date of survey, and state of residence to account for differences in policies across geographic areas.^13^ Logit and probit models yielded similar marginal effects, but ordinary least squares estimates are presented for ease of interpretation (see eTable 3 and eTable 4 in the Supplement). Two-sided *P* values calculated with the *t* test for the ordinary least squares coefficient estimate are reported, with *P* < .05 considered statistically significant. The ordinary least squares coefficient estimate represents the mean difference between the given group and a reference group. Data analysis was performed with Stata statistical software version 15 (StataCorp). Data analysis was performed in April 2020.

## Results 

 The survey included 5198 individuals (mean [SD] age, 48 [18] years), including 2336 men (45%), 3759 white individuals (72%), 830 African American or black individuals (16%), and 609 Hispanic individuals (12%). Table 1 compares the survey sample’s demographic characteristics with those in the US as a whole. The survey sample is similar to the overall US population in terms of sex (female 55% vs 52%), age distribution (18-29 years, 20% vs 27%; 30-39 years, 16% vs 19%; 40-49 years, 14% vs 21%; 50-59 years, 16% vs 20%; 60-69 years, 18% vs 14%), race/ethnicity (white, 72% vs 77%; black or African American, 16% vs 13%; Hispanic, 12% vs 18%), and employment characteristics (employed, 53% vs 48%; unemployed, 14% vs 2%), although the percentage of college graduates in the study sample is higher than that in the country as a whole (51% vs 32%).

**Table 1.** Demographic Characteristics: Survey Sample vs US Census^a^

Characteristic	Survey sample, participants, No. (%)			US population, %
	Inside hotspot^b^ (n = 1082)	Outside hotspot^b^ (n = 4116)	Total (N = 5198)	
Sex				
Male	487 (45)	1849 (45)	2336 (45)	48
Female	591 (55)	2259 (55)	2850 (55)	52
Age, y				
18-29	240 (22)	813 (20)	1053 (20)	27
30-39	254 (23)	599 (14)	853 (16)	19
40-49	194 (18)	514 (12)	708 (14)	21
50-59	133 (12)	722 (18)	855 (16)	20
60-69	133 (12)	828 (20)	961 (18)	14
Race/ethnicity				
White	775 (72)	2984 (73)	3759 (72)	77
Black or African American	178 (16)	652 (16)	830 (16)	13
Hispanic	129 (12)	480 (12)	609 (12)	18
Annual gross household income				
<$25 000	188 (17)	941 (23)	1129 (22)	19
$25 000-$49 999	199 (18)	850 (21)	1049 (20)	21
$50 0000-$74 999	165 (15)	758 (18)	923 (18)	17
$75 000-$99 999	162 (15)	576 (14)	738 (14)	12
≥$100 000	368 (34)	991 (24)	1359 (26)	30
Employment status				
Employed	685 (63)	2083 (51)	2768 (53)	48
Unemployed	127 (12)	624 (15)	751 (14)	2
College degree	674 (62)	1987 (48)	2661 (51)	32

^a^ The table shows summary statistics of demographic characteristics of survey respondents alongside data from the US as a whole (the fifth column). The source for the fifth column is the US Census Bureau and Current Population Survey, 2018.

^b^ The following areas were considered hotspots: New York, New York; New Orleans, Louisiana; Detroit, Michigan; and Seattle, Washington. Residence in a hotspot was coded as a binary variable, which was equal to 1 if the respondent lived in any of the 4 hotspot areas, or 0 otherwise.

Table 2 shows data for the measures of disease exposure, knowledge, beliefs, and behaviors. Two hundred fourteen people (4.0%) reported being infected with COVID-19, and 1208 (23.0%) reported knowing someone infected with COVID-19. Knowledge about the spread of COVID-19 was very high. Four thousand two hundred fifteen people (83.0%) believed that COVID-19 can be contracted from a contaminated surface, and 4404 (87.0%) knew all 3 symptoms of COVID-19 (fever, cough, and difficulty in breathing). Very few people (553 participants [10.9%]) associated COVID-19 with sexual transmission. Only 257 people (5.1%) believed that COVID-19 is a hoax.

**Table 2.** Survey Summary Statistics of Major Outcomes: Full Sample and by Hotspot

Variable	Participants, No. (%)^a^		
	Total (N = 5198)	Inside hotspot (n = 1082)	Outside hotspot (n = 4116)
Knowledge about spread			
Through respiratory droplets	4339 (86.0)	875 (83.0)	3464 (86.0)
Through close contact	3932 (78.0)	801 (76.0)	3131 (78.0)
Through a contaminated surface	4215 (83.0)	853 (81.0)	3362 (84.0)
Spread without showing symptoms	4778 (94.0)	978 (93.0)	3800 (95.0)
Through unprotected sex	553 (10.9)	145 (13.7)	408 (10.2)
The virus is a hoax	257 (5.1)	86 (8.2)	171 (4.3)
Knowledge about symptoms			
Fever	4758 (94.0)	972 (92.0)	3786 (94.0)
Cough	4770 (94.0)	968 (92.0)	3802 (95.0)
Difficulty in breathing	4681 (92.0)	951 (90.0)	3730 (93.0)
All 3 symptoms	4404 (87.0)	887 (84.0)	3517 (88.0)
Any other symptoms reported	512 (10.0)	131 (12.0)	381 (9.0)
Reports COVID-19 infection			
Self	214 (4.0)	74 (7.0)	140 (3.0)
Other	1208 (23.0)	434 (40.0)	774 (19.0)
Subjective likelihood, mean (SD), score			
Likelihood of getting sick from COVID-19 in next month, score 0-10	3.9 (2.5)	4.1 (2.5)	3.9 (2.5)
No. of community members likely to get sick from COVID-19 in next month, score 0-100	37.6 (30.2)	46.7 (30.5)	35.2 (29.7)
Behaviors, mean (SD), No. of times			
Handwashing in last 24 h	13.2 (13.5)	13.0 (13.0)	13.2 (13.6)
Left home in 3 d	2.5 (4.0)	2.8 (4.6)	2.4 (3.8)
Frequent hospital use	675 (13.0)	180 (17.0)	495 (12.0)
Ease of accessing testing, mean (SD), score 0-10^b^	4.2 (2.9)	4.3 (3.1)	4.2 (2.9)

Abbreviation: COVID-19, coronavirus disease 2019.

^a^ No. is the number eligible to answer. A small number of people did not answer each question.

^b^ 0 = extremely difficult; 10 = extremely easy.

**Figure 1.**
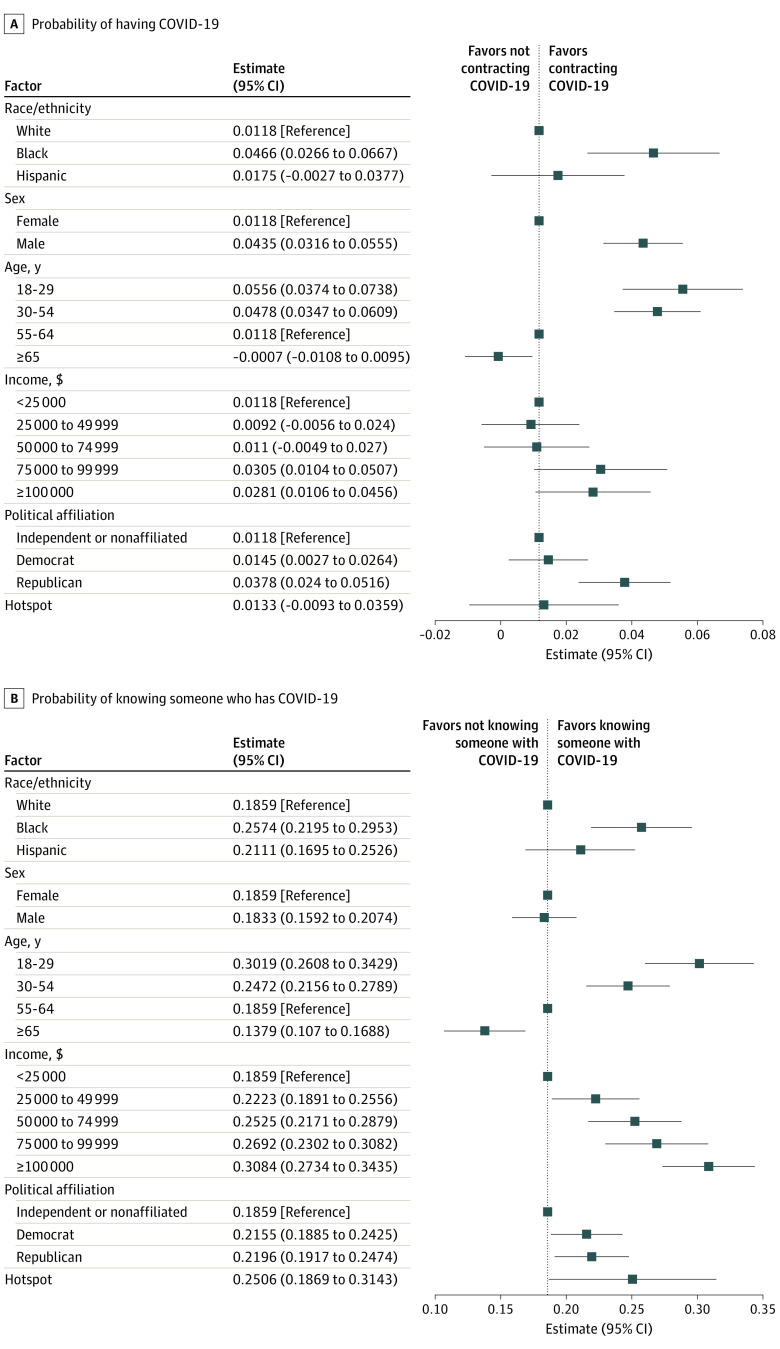
Association of Demographic Characteristics, Socioeconomic Status, Geographic Location, and Political Orientation With the Probability of Having Coronavirus Disease 2019 (COVID-19) or Knowing Someone Who Does

Figure 1A shows the differential associations of demographic characteristics, socioeconomic status, geographic location, and political orientation on the probability of testing positive for COVID-19 or knowing someone who did. African American respondents were 3.5 percentage points (95% CI, 1.5 to 5.5 percentage points; *P* = .001) more likely than white respondents to report being infected with COVID-19 as were men compared with women (3.2 percentage points; 95% CI, 2.0 to 4.4 percentage points; *P* < .001). Those who affiliate with the Republican party were 2.6 percentage points (95% CI, 1.2 to 4.0 percentage points; *P* < .001) more likely to report infection than were political independents.

Turning to the question on infection of acquaintances in Figure 1B, respondents in a hotspot area were more likely to know somehow who tested positive for COVID-19 than were people living in other areas (6.5 percentage points; 95% CI, 0.10 to 12.8 percentage points; *P* < .05). African American respondents were far more likely to report knowing someone who tested positive for COVID-19 than were white respondents (7.2 percentage points; 95% CI, 3.4 to 10.9 percentage points; *P* < .001). More young people reported knowing someone who tested positive for COVID-19 (coefficient on age 18-29 years, 11.6 percentage points; 95% CI, 7.5 to 15.7 percentage points; *P* < .001) as did people with higher incomes (coefficient on earning ≥$100 000, 12.3 percentage points; 95% CI, 8.7 to 15.8 percentage points; *P* < .001). Despite the partisan nature of reaction to the pandemic, Democrats and Republicans were approximately equally likely to know someone who tested positive for COVID-19 than nonaffiliated or independent individuals.

**Figure 2.**
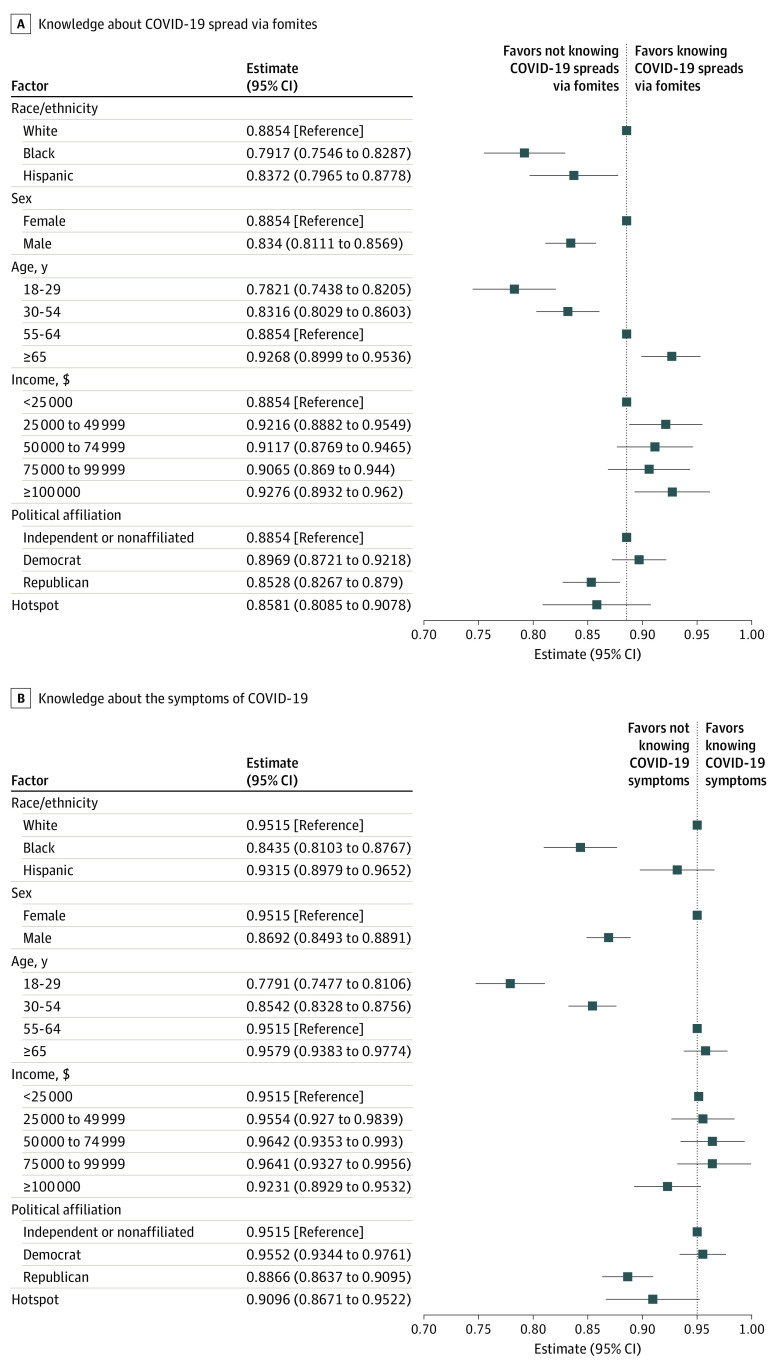
Factors Associated With Knowledge About the Symptoms and Spread of Coronavirus Disease 2019 (COVID-19)

Figure 2 shows the factors associated with higher knowledge about the spread of COVID-19. The largest gaps in knowledge were observed among racial/ethnic minorities, men, and young people (aged <55 years). African American respondents were 9.4 percentage points less likely than white respondents to know that a person can become infected by touching a contaminated surface (95% CI, −13.1 to −5.7 percentage points; *P* < .001). Hispanic respondents were also 4.8 percentage points less likely than white respondents to understand fomite spread (95% CI, −8.9 to −0.77 percentage points; *P* = .02). Male respondents were 5.1 percentage points (95% CI, −7.4 to −2.9 percentage points; *P* < .001) less likely to know this information than female respondents, and the youngest age group (aged <30 years) was 10.3 percentage points (95% CI, 14.1 to 6.5 percentage points; *P* < .001) less likely to be aware than the reference age group (55-64 years). There were also observed differences in knowledge about COVID-19 by political affiliation and income. People with higher incomes reported knowing more about fomite spread than people with lower incomes (difference between annual income ≥$100 000 and annual income <$25 000, 4.2 percentage points; 95% CI, 0.78 to 7.7 percentage points; *P* = .02), whereas Republicans were less likely to report knowing about fomite spread (difference between Republicans and nonaffiliated or independent individuals, 3.3 percentage points; 95% CI, −5.8 to −0.64 percentage points; *P* = .02). eTable 5 in the Supplement shows additional results on asymptomatic transmission, as well as transmission via respiratory droplets or contact within an infected person. The results are overall consistent with those reported in the main text.

Very similar associations were found with respect to knowledge of COVID-19 symptoms. African American respondents were 10.8 percentage points (95% CI, −14.1 to −7.5 percentage points; *P* < .001) less likely than non-Hispanic white respondents to know that cough, fever, and shortness of breath are symptoms of COVID-19. Similarly, people aged 18 to 29 years were 17.2 percentage points (95% CI, −20.4 to −14.1 percentage points, *P* < .001) less likely than people aged 55 to 64 years to know the symptoms of COVID-19.

**Figure 3.**
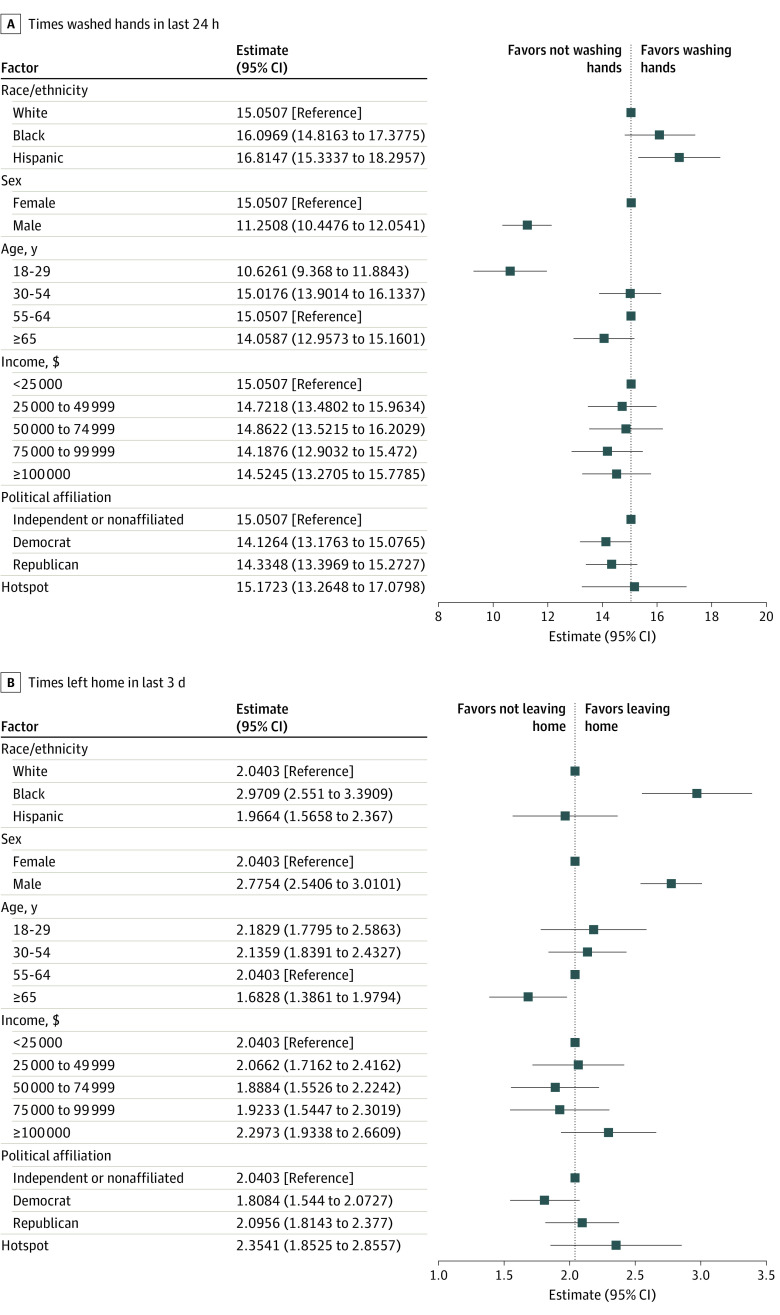
Factors Associated With Handwashing and Leaving the House

Figure 3 shows differences in handwashing and leaving the house for the same groups. With respect to handwashing, the largest differences were between men and women and between younger and older groups. Men washed their hands 3.8 times less per 24 hours than women (95% CI, −4.6 to −3.0 times; *P* < .001) and people aged 18 to 29 years washed their hands 4.4 times less per 24 hours than older individuals (95% CI, −5.7 to −3.2 times; *P* < .001). Hispanic respondents, on the other hand, washed their hands more frequently than white respondents in a 24-hour period (1.8 times more; 95% CI, 0.3 to 3.2 times; *P* = .02). Men were also more likely than women to leave the house frequently (0.74 times; 95% CI, 0.5 to 1.0 times; *P* < .001), as were African American respondents compared with white respondents (0.93 times; 95% CI, 0.5 to 1.4 times; *P* < .001). Older respondents (aged ≥65 years) were less likely than younger respondents to leave their homes frequently (−0.36 times; 95% CI, −0.7 to −0.1 times; *P* = .02). Neither frequent handwashing nor staying indoors differed by income, residence in a hotspot area, or political orientation.

## Discussion

For society to control the spread of COVID-19, it is essential that public health officials know which groups are most heavily affected by the disease and that all individuals have access to trusted guidance on how to protect themselves and their communities from infection. Given the increased susceptibility to COVID-19 among low-income households and racial/ethnic minorities, it is important to determine how accurate knowledge and public health behavior regarding COVID-19 is among these groups. Furthermore, there is concern that a partisan divide in COVID-19–associated news coverage has weakened the effectiveness of public health messaging overall.

This study used national survey data conducted in the first few weeks of the COVID-19 pandemic in the US to discern reported exposure, knowledge, and behaviors regarding its spread. The study reached 3 conclusions. First, similar to what has been documented in the media, African American respondents were more likely to report COVID-19 exposure or to know someone who has been infected. Reported COVID-19 exposure was also higher for men and younger age groups.

Second, although knowledge about the novel virus and how to reduce its spread was high, there were important differences across groups that map to behaviors. In the population as a whole, more than 80% of people knew that they can contract COVID-19 from a contaminated surface, and even higher shares knew the 3 cardinal signs of having COVID-19. Such high levels of knowledge for a novel disease testify to the role of public health officials and the news media in explaining the basics of disease spread. However, African American respondents, men, and younger people, the very same groups that report higher COVID-19 exposure, had less accurate knowledge than white respondents, women, and older individuals. The gaps are consistently largest for African American respondents, who were less likely to know about fomite transmission, asymptomatic transmission, respiratory droplet transmission, or the 3 common symptoms of COVID-19.

Third, there were important differences in reported behavior. African Americans, men, and younger individuals were more likely to leave their homes. Some of the difference in behavior may be associated with social circumstances. For example, African American individuals are less likely to be able to telecommute and more likely to work in the service sector and use public transportation than other racial/ethnic groups (23% among African American individuals vs 15% among Hispanic individuals and 7% among white individuals).^14^ Strikingly, knowledge and behaviors were closely related; groups in which behaviors put people more at risk for disease were also groups in which knowledge of appropriate behaviors are weakest. These findings suggest that greater efforts to communicate risk and safe practices to racial/ethnic minorities and younger people may be particularly crucial moving forward.

### Limitations

The study has several limitations. First, knowledge and behaviors associated with COVID-19 are changing rapidly, and it is possible that the findings here will be less applicable over time. Second, the survey was conducted by internet, which may bias the results toward those with access to Wi-Fi. Third, the results on reported COVID-19 infection did not specify whether the person had been tested for the disease.

## Conclusions

These findings confirm anecdotal reports of concerning gaps in reported prevalence and knowledge regarding COVID-19 across different demographic characteristics in the US population. More effort will be needed to address the knowledge and behavior gaps between white and African American individuals, between men and women, and between older and younger populations.

## Notes

Supplement. **eAppendix.** Survey Question Wording **eTable 1.** Summary Statistics **eTable 2.** Linear Regression Analysis on Reported COVID-19+ Infection, Knowledge, and Behaviors **eTable 3.** Logit Regression Analysis on Reported COVID-19+ Infection, Knowledge, and Behaviors **eTable 4.** Probit Regression Analysis on Reported COVID-19+ Infection, Knowledge, and Behaviors **eTable 5.** Linear Regression Analysis on Knowledge and Risks
