# Supplementary material for: Disparities in Coronavirus 2019 Reported Incidence, Knowledge, and Behavior Among US Adults
Source: JAMA Netw Open. 2020 Jun 18;3(6):e2012403. doi: 10.1001/jamanetworkopen.2020.12403 (PMC7303811; doi:10.1001/jamanetworkopen.2020.12403)
Supplement: Supplement. — eAppendix. Survey Question Wording eTable 1. Summary Statistics eTable 2. Linear Regression Analysis on Reported COVID-19+ Infection, Knowledge, and Behaviors eTable 3. Logit Regression Analysis on Reported COVID-19+ Infection, Knowledge, and Behaviors eTable 4. Probit Regression Analysis on Reported COVID-19+ Infection, Knowledge, and Behaviors eTable 5. Linear Regression Analysis on Knowledge and Risks [file jamanetwopen-3-e2012403-s001.pdf]

Supplementary Online Content

Alsan M, Stantcheva S, Yang D, Cutler D. Disparities in coronavirus 2019 reported incidence, knowledge, and behavior among US adults. *JAMA Netw Open*. 2020;3(6):e2012403.
doi:10.1001/jamanetworkopen.2020.12403

eAppendix. Survey Question Wording
eTable 1. Summary Statistics
eTable 2. Linear Regression Analysis on Reported COVID-19+ Infection, Knowledge, and Behaviors
eTable 3. Logit Regression Analysis on Reported COVID-19+ Infection, Knowledge, and Behaviors
eTable 4. Probit Regression Analysis on Reported COVID-19+ Infection, Knowledge, and Behaviors
eTable 5. Linear Regression Analysis on Knowledge and Risks

This supplementary material has been provided by the authors to give readers additional information about their work.

© 2020 Alsan M et al. *JAMA Network Open.*

eAppendix. Survey Question Wording

1. Can someone with COVID-19 infect others without feeling sick or without showing any symptoms? (Yes or No)

2. How does COVID-19 spread? (Select all that apply)
▢ When in close contact with an infected person (within about 6 feet). (1)
▢ Through respiratory droplets produced when an infected person coughs or sneezes. (2)
▢ Touch a contaminated surface and then touching your eyes nose or mouth. (3)
▢ Though unprotected sex. (4)
▢ The virus is a hoax. (5)

3. In the last 24 hours, how many times have you washed your hands? (Please enter numbers only)

4. In the last 3 days, how many times have you left your residence? (Please enter numbers only)

5. Have you already gotten infected with the Coronavirus COVID-19?

6. How many people do you know personally who have become seriously ill from COVID-19 or died from COVID-19 so far?

7. How likely do you think you are to get sick from COVID-19 in the next month?

8. Take 100 randomly selected people (not including you) who live in your town/city. How many, do you think, will get sick from COVID-19 in the next month?

9. From the list below, select the top 3 symptoms associated with COVID-19?
▢ Fever (1)
▢ Dry eyes (2)
▢ Skin rash (3)
▢ Cough (4)
▢ Difficulty breathing (5)
▢ Swollen legs (6)
▢ Acid Reflux (7)
▢ Stomach ache (8)
▢ Watery eyes (9)

© 2020 Alsan M et al. *JAMA Network Open.*

eTable 1. Summary Statistics[a]

Variable	Mean	SD	Min.	Max.	N
(B) Gender: 1 if R is Male.	0.45	0.498	0	1	5186
R's calculated age	48.337	18.12	18	120	5198
(B) Citizenship: 1 if R is a U.S. Citizen.	0.984	0.126	0	1	5196
(B) Income: household income level categorized by the percentile.	1.852	0.83	1	4	5198
(B) Income: 1 if R's household income level is greater than the 50th percentile.	0.403	0.491	0	1	5198
(B) Income: 1 if R's household income level is greater than the 75th percentile.	0.118	0.323	0	1	5198
(B) Education: 1 if R has a high school or secondary school diploma.	0.797	0.402	0	1	5198
(B) Education: 1 if R received some college education.	0.64	0.48	0	1	5198
(B) Education: 1 if R has a 2-year, or 4-year college diploma.	0.512	0.5	0	1	5198
(B) Insurance: 1 if R has a health insurance.	0.899	0.301	0	1	5198
(B) Any Health Condition	0.23	0.421	0	1	5198
(B) Hotspot: 1 if R lives in a "hotspot".	0.208	0.406	0	1	5198
Survey Duration (in minutes)	26.247	15.789	2.4	118.833	5198
R's willingness in taking risks in general	5.711	2.567	0	10	5198
R's time preference level	6.952	2.011	0	10	5198
(B) 1 if R is a Democrat.	0.387	0.487	0	1	5198
(B) 1 if R is a Republican.	0.291	0.454	0	1	5198
(B) 1 if R is White/Caucasian.	0.723	0.447	0	1	5198
(B) 1 if R is Black/African-American.	0.16	0.366	0	1	5198
(B) 1 if R is Hispanic.	0.117	0.322	0	1	5198

Abbreviation: R, respondent.
[a] The table reports summary statistics of major demographic characteristics. (B) indicates that the variable is binary.

© 2020 Alsan M et al. *JAMA Network Open.*

eTable 2. Linear Regression Analysis on Reported COVID-19+ Infection, Knowledge, and Behaviors[a]

	Respondent is COVID-19+[b]	Acquaintance is COVID-19+[c]	Contaminated Surface[d]	All Three Symptoms[e]	Handwashing 24 Hours[f]	Left House 3 Days[f]
African-American/Black	0.035***	0.072***	-0.094***	-0.108***	1.046	0.931***
	(0.010)	(0.019)	(0.019)	(0.017)	(0.653)	(0.214)
Hispanic	0.006	0.025	-0.048**	-0.020	1.764**	-0.074
	(0.010)	(0.021)	(0.021)	(0.017)	(0.756)	(0.204)
Male	0.032***	-0.003	-0.051***	-0.082***	-3.800***	0.735***
	(0.006)	(0.012)	(0.012)	(0.010)	(0.410)	(0.120)
Age 18 - 29	0.044***	0.116***	-0.103***	-0.172***	-4.425***	0.143
	(0.009)	(0.021)	(0.020)	(0.016)	(0.642)	(0.206)
Age 30 - 54	0.036***	0.061***	-0.054***	-0.097***	-0.033	0.096
	(0.007)	(0.016)	(0.015)	(0.011)	(0.569)	(0.151)
Age 65 or above	-0.012**	-0.048***	0.041***	0.006	-0.992*	-0.358**
	(0.005)	(0.016)	(0.014)	(0.010)	(0.562)	(0.151)
USD 25,000 to 49,999	-0.003	0.036**	0.036**	0.004	-0.329	0.026
	(0.008)	(0.017)	(0.017)	(0.015)	(0.633)	(0.179)
USD 50,000 to 74,999	-0.001	0.067***	0.026	0.013	-0.189	-0.152
	(0.008)	(0.018)	(0.018)	(0.015)	(0.684)	(0.171)
USD 75,000 to 99,999	0.019*	0.083***	0.021	0.013	-0.863	-0.117
	(0.010)	(0.020)	(0.019)	(0.016)	(0.655)	(0.193)
USD 100,000 or above	0.016*	0.123***	0.042**	-0.028*	-0.526	0.257
	(0.009)	(0.018)	(0.018)	(0.015)	(0.640)	(0.185)
Democrat	0.003	0.030**	0.011	0.004	-0.924*	-0.232*
	(0.006)	(0.014)	(0.013)	(0.011)	(0.485)	(0.135)
Republican	0.026***	0.034**	-0.033**	-0.065***	-0.716	0.055
	(0.007)	(0.014)	(0.013)	(0.012)	(0.479)	(0.144)
Hotspot[g]	0.001	0.065**	-0.027	-0.042*	0.122	0.314
	(0.012)	(0.033)	(0.025)	(0.022)	(0.973)	(0.256)
Health Insurance	0.016*	0.016	0.066***	0.063***	0.040	-0.266
	(0.009)	(0.020)	(0.021)	(0.019)	(0.656)	(0.221)
High Health Risk[h]	0.066***	0.100***	-0.059***	-0.112***	-0.744*	0.376**
	(0.008)	(0.014)	(0.013)	(0.012)	(0.418)	(0.150)
Risk Preference[i]	0.009***	0.017***	-0.012***	-0.014***	0.010	0.118***
	(0.001)	(0.002)	(0.002)	(0.002)	(0.088)	(0.022)
State Fixed Effects	Yes	Yes	Yes	Yes	Yes	Yes
Survey Date Fixed Effects	Yes	Yes	Yes	Yes	Yes	Yes
No. Obs.	5181	5171	5060	5056	5186	5186
Wald Black=Hispanic	0.017	0.060	0.063	0.000	0.420	0.000
Mean of Dept. Var.	0.041	0.233	0.831	0.869	13.175	2.508

[a] Table estimated using OLS. Results are based on the Harvard-Dynata survey limited to the United States. Not reported but included in each specification are survey date and state fixed effects. Robust standard errors in parentheses. * p < .10 ** p < .05 ***p < .001.
[b] Respondent COVID-19+ is a binary variable equal to 1 if the respondent reports contracting COVID-19 already.
[c] Acquaintance is COVID-19+ is a binary variable equal to 1 if the respondent knows anyone with COVID-19.
[d] Contaminated Surface is a binary variable equal to 1 if the respondent knows that COVID-19 can be transmitted by touching a contaminated surface.
[e] All Three Symptoms is a binary variable equal to 1 if the respondent knows that cough, fever and shortness of breath are symptoms of COVID-19.
[f] Handwashing and Leave House are binary variables equal to 1 if the respondent is in the top 50th and 75th percentile of either activity, (more than 10 and 3 times) respectively.
[g] Hotspot is a binary variable equal to 1 for New York City, New Orleans, Detroit, and Seattle.
[h] High health risk is a binary variable equal to 1 if the respondent endorses a diagnosis of chronic lung disease, cardiovascular disease or diabetes.
[i] Risk preference is a categorical variable from 0-10 which refers to a respondent’s preference for risk, in general.

© 2020 Alsan M et al. *JAMA Network Open.*

eTable 3. Logit Regression Analysis on Reported COVID-19+ Infection, Knowledge, and Behaviors[a]

	Respondent is COVID-19+[b]	Acquaintance is COVID-19+[c]	Contaminated Surface[d]	All Three Symptoms[e]	Handwashing 24 Hours[f]	Left House 3 Days[f]
African-American/Black	0.944***	0.418***	-0.608***	-0.932***	-0.047	0.673***
	(0.229)	(0.110)	(0.117)	(0.138)	(0.094)	(0.112)
Hispanic	0.298	0.181	-0.342***	-0.276*	0.007	0.078
	(0.249)	(0.123)	(0.131)	(0.151)	(0.106)	(0.142)
Male	0.901***	-0.036	-0.401***	-0.858***	-0.614***	0.651***
	(0.175)	(0.078)	(0.088)	(0.103)	(0.065)	(0.086)
Age 18 - 29	1.356***	0.680***	-0.704***	-1.805***	-0.713***	0.197
	(0.339)	(0.129)	(0.142)	(0.196)	(0.106)	(0.136)
Age 30 - 54	1.066***	0.370***	-0.426***	-1.217***	-0.115	0.044
	(0.316)	(0.109)	(0.125)	(0.182)	(0.086)	(0.119)
Age 65 or above	-0.682	-0.374***	0.502***	0.098	-0.165*	-0.319**
	(0.430)	(0.126)	(0.154)	(0.224)	(0.091)	(0.133)
USD 25,000 to 49,999	-0.018	0.241**	0.264**	0.025	0.018	-0.054
	(0.292)	(0.118)	(0.124)	(0.151)	(0.093)	(0.127)
USD 50,000 to 74,999	0.000	0.438***	0.187	0.167	-0.010	-0.255*
	(0.299)	(0.123)	(0.131)	(0.163)	(0.098)	(0.140)
USD 75,000 to 99,999	0.662**	0.548***	0.137	0.123	-0.068	0.022
	(0.277)	(0.128)	(0.139)	(0.176)	(0.106)	(0.141)
USD 100,000 or above	0.391	0.770***	0.327**	-0.219	-0.008	0.134
	(0.248)	(0.114)	(0.129)	(0.149)	(0.095)	(0.124)
Democrat	0.056	0.193**	0.095	0.045	-0.080	-0.139
	(0.207)	(0.088)	(0.100)	(0.120)	(0.072)	(0.097)
Republican	0.520**	0.192**	-0.227**	-0.583***	0.039	0.030
	(0.208)	(0.095)	(0.103)	(0.120)	(0.077)	(0.101)
Hotspot[g]	-0.217	0.221	-0.215	-0.504*	0.082	0.316
	(0.332)	(0.169)	(0.211)	(0.289)	(0.150)	(0.205)
Health Insurance	0.388	0.081	0.418***	0.555***	0.038	-0.234*
	(0.298)	(0.129)	(0.121)	(0.138)	(0.103)	(0.128)
High Health Risk[h]	1.368***	0.603***	-0.429***	-0.960***	-0.088	0.156*
	(0.162)	(0.082)	(0.092)	(0.104)	(0.071)	(0.092)
Risk Preference[i]	0.276***	0.115***	-0.089***	-0.150***	0.004	0.082***
	(0.043)	(0.016)	(0.018)	(0.023)	(0.012)	(0.017)
State Fixed Effects	Yes	Yes	Yes	Yes	Yes	Yes
Survey Date Fixed Effects	Yes	Yes	Yes	Yes	Yes	Yes
No. Obs	4961	5150	5012	5022	5185	5148
Wald Black=Hispanic	0.018	0.082	0.059	0.000	0.651	0.000
Mean of Dept. Var.	0.041	0.233	0.831	0.869	0.390	0.159

[a] Table estimated using logit. Results are based on the Harvard-Dynata survey limited to the United States. Not reported but included in each specification are survey date and state fixed effects. Robust standard errors in parentheses. * p < .10 ** p < .05 ***p < .001.
[b] Respondent COVID-19+ is a binary variable equal to 1 if the respondent reports contracting COVID-19 already.
[c] Acquaintance is COVID-19+ is a binary variable equal to 1 if the respondent knows anyone with COVID-19.
[d] Contaminated Surface is a binary variable equal to 1 if the respondent knows that COVID-19 can be transmitted by touching a contaminated surface.
[e] All Three Symptoms is a binary variable equal to 1 if the respondent knows that cough, fever and shortness of breath are symptoms of COVID-19.
[f] Handwashing and Leave House are binary variables equal to 1 if the respondent is in the top 50th and 75th percentile of either activity, (more than 10 and 3 times) respectively.
[g] Hotspot is a binary variable equal to 1 for New York City, New Orleans, Detroit, and Seattle.
[h] High health risk is a binary variable equal to 1 if the respondent endorses a diagnosis of chronic lung disease, cardiovascular disease or diabetes.
[i] Risk preference is a categorical variable from 0-10 which refers to a respondent's preference for risk, in general.

© 2020 Alsan M et al. *JAMA Network Open.*

eTable 4. Probit Regression Analysis on Reported COVID-19+ Infection, Knowledge, and Behaviors[a]

	Respondent is COVID-19+[b]	Acquaintance is COVID-19+[c]	Contaminated Surface[d]	All Three Symptoms[e]	Handwashing 24 Hours[f]	Left House 3 Days[f]
African-American/Black	0.475***	0.247***	-0.354***	-0.508***	-0.030	0.376***
	(0.107)	(0.064)	(0.066)	(0.074)	(0.058)	(0.064)
Hispanic	0.124	0.104	-0.198***	-0.130	0.004	0.046
	(0.118)	(0.072)	(0.074)	(0.083)	(0.065)	(0.078)
Male	0.422***	-0.025	-0.219***	-0.472***	-0.377***	0.358***
	(0.081)	(0.045)	(0.049)	(0.056)	(0.040)	(0.047)
Age 18 - 29	0.615***	0.399***	-0.381***	-0.931***	-0.439***	0.105
	(0.148)	(0.073)	(0.077)	(0.097)	(0.065)	(0.075)
Age 30 - 54	0.490***	0.208***	-0.219***	-0.612***	-0.072	0.024
	(0.136)	(0.062)	(0.067)	(0.088)	(0.053)	(0.065)
Age 65 or above	-0.311*	-0.207***	0.262***	0.037	-0.105*	-0.177**
	(0.174)	(0.069)	(0.079)	(0.106)	(0.056)	(0.071)
USD 25,000 to 49,999	-0.031	0.137**	0.149**	0.015	0.010	-0.028
	(0.131)	(0.067)	(0.069)	(0.081)	(0.057)	(0.069)
USD 50,000 to 74,999	-0.028	0.254***	0.104	0.115	-0.006	-0.141*
	(0.136)	(0.070)	(0.073)	(0.087)	(0.060)	(0.075)
USD 75,000 to 99,999	0.338***	0.318***	0.086	0.102	-0.042	0.013
	(0.129)	(0.074)	(0.078)	(0.093)	(0.065)	(0.078)
USD 100,000 or above	0.194*	0.447***	0.189***	-0.089	-0.005	0.073
	(0.118)	(0.066)	(0.071)	(0.080)	(0.058)	(0.069)
Democrat	-0.008	0.116**	0.056	0.014	-0.049	-0.074
	(0.096)	(0.051)	(0.055)	(0.063)	(0.044)	(0.053)
Republican	0.224**	0.109**	-0.117**	-0.318***	0.023	0.019
	(0.097)	(0.054)	(0.057)	(0.065)	(0.047)	(0.056)
Hotspot[g]	-0.140	0.139	-0.114	-0.251*	0.050	0.170
	(0.147)	(0.099)	(0.114)	(0.146)	(0.092)	(0.111)
Health Insurance	0.197	0.040	0.240***	0.309***	0.023	-0.130*
	(0.134)	(0.073)	(0.070)	(0.077)	(0.063)	(0.072)
High Health Risk[h]	0.682***	0.345***	-0.233***	-0.509***	-0.055	0.080
	(0.078)	(0.048)	(0.051)	(0.056)	(0.044)	(0.051)
Risk Preference[i]	0.123***	0.065***	-0.049***	-0.075***	0.003	0.045***
	(0.019)	(0.009)	(0.010)	(0.012)	(0.008)	(0.009)
State Fixed Effects	Yes	Yes	Yes	Yes	Yes	Yes
Survey Date Fixed Effects	Yes	Yes	Yes	Yes	Yes	Yes
No. Obs	4961	5150	5012	5022	5185	5148
Wald Black=Hispanic	0.007	0.073	0.056	0.000	0.644	0.000
Mean of Dept. Var.	0.041	0.233	0.831	0.869	0.390	0.159

[a] Table estimated using probit. Results are based on the Harvard-Dynata survey limited to the United States. Not reported but included in each specification are survey date and state fixed effects. Robust standard errors in parentheses. * p < .10 ** p < .05 ***p < .001.

[b] Respondent COVID-19+ is a binary variable equal to 1 if the respondent reports contracting COVID-19 already.

[c] Acquaintance is COVID-19+ is a binary variable equal to 1 if the respondent knows anyone with COVID-19.

[d] Contaminated Surface is a binary variable equal to 1 if the respondent knows that COVID-19 can be transmitted by touching a contaminated surface.

[e] All Three Symptoms is a binary variable equal to 1 if the respondent knows that cough, fever and shortness of breath are symptoms of COVID-19.

[f] Handwashing and Leave House are binary variables equal to 1 if the respondent is in the top 50th and 75th percentile of either activity, (more than 10 and 3 times) respectively.

[g] Hotspot is a binary variable equal to 1 for New York City, New Orleans, Detroit, and Seattle.

[h] High health risk is a binary variable equal to 1 if the respondent endorses a diagnosis of chronic lung disease, cardiovascular disease or diabetes.

[i] Risk preference is a categorical variable from 0-10 which refers to a respondent's preference for risk, in general.

© 2020 Alsan M et al. *JAMA Network Open.*

eTable 5. Linear Regression Analysis on Knowledge and Risks[a]

	Resp. Droplets[b]	Close Contact Spread[c]	Asymptomatic Spread[d]	Placebo Symptoms[e]	COVID-19 Hoax[f]	HH. Hosp. Use[g]
African-American/Black	-0.119***	-0.061***	-0.065***	0.083***	0.028**	0.082***
	(0.018)	(0.020)	(0.014)	(0.015)	(0.012)	(0.016)
Hispanic	-0.043**	-0.051**	-0.011	0.012	0.002	0.012
	(0.020)	(0.022)	(0.013)	(0.016)	(0.012)	(0.017)
Male	-0.028***	-0.033**	-0.015**	0.063***	0.015**	0.023**
	(0.010)	(0.013)	(0.007)	(0.009)	(0.007)	(0.010)
Age 18 - 29	-0.176***	-0.084***	-0.071***	0.150***	0.062***	0.179***
	(0.018)	(0.022)	(0.013)	(0.015)	(0.011)	(0.016)
Age 30 - 54	-0.075***	-0.021	-0.037***	0.083***	0.057***	0.091***
	(0.012)	(0.017)	(0.008)	(0.009)	(0.007)	(0.011)
Age 65 or above	0.026**	0.066***	0.001	-0.006	-0.013***	-0.035***
	(0.011)	(0.017)	(0.006)	(0.008)	(0.005)	(0.010)
USD 25,000 to 49,999	0.057***	0.034*	0.035***	-0.001	-0.014	-0.016
	(0.016)	(0.019)	(0.010)	(0.013)	(0.009)	(0.014)
USD 50,000 to 74,999	0.055***	0.037*	0.022**	-0.006	-0.012	-0.010
	(0.017)	(0.019)	(0.011)	(0.013)	(0.009)	(0.015)
USD 75,000 to 99,999	0.056***	0.005	0.003	0.007	-0.009	-0.006
	(0.018)	(0.021)	(0.013)	(0.015)	(0.010)	(0.016)
USD 100,000 or above	0.036**	0.007	0.003	0.036***	0.021**	0.008
	(0.017)	(0.020)	(0.012)	(0.014)	(0.010)	(0.015)
Democrat	0.018	0.029**	0.017**	-0.005	-0.011	0.028***
	(0.011)	(0.014)	(0.008)	(0.009)	(0.007)	(0.010)
Republican	-0.042***	-0.013	-0.003	0.055***	0.018**	0.064***
	(0.012)	(0.015)	(0.008)	(0.011)	(0.008)	(0.011)
Hotspot [h]	-0.019	-0.027	-0.032*	0.044**	0.036***	0.034
	(0.025)	(0.029)	(0.018)	(0.019)	(0.012)	(0.021)
Health Insurance	0.059***	0.069***	0.069***	-0.042**	0.007	0.074***
	(0.020)	(0.023)	(0.016)	(0.017)	(0.012)	(0.015)
High Health Risk[i]	-0.081***	-0.036**	-0.015*	0.099***	0.042***	0.189***
	(0.012)	(0.014)	(0.008)	(0.011)	(0.008)	(0.012)
Risk Preference[j]	-0.010***	-0.008***	-0.002*	0.013***	0.006***	0.018***
	(0.002)	(0.003)	(0.001)	(0.002)	(0.001)	(0.002)
State Fixed Effects	Yes	Yes	Yes	Yes	Yes	Yes
Survey Date Fixed Effects	Yes	Yes	Yes	Yes	Yes	Yes
No. Obs	5060	5060	5055	5056	5060	5183
Wald Black=Hispanic	0.001	0.694	0.001	0.000	0.068	0.001
Mean of Dept. Var.	0.856	0.775	0.943	0.101	0.051	0.130

[a] Table estimated using OLS. Results are based on the Harvard-Dynata survey limited to the United States. Not reported but included in each specification are survey date and state fixed effects. Robust standard errors in parentheses. * p < .10 ** p < .05 ***p < .001

[b] Respiratory Droplets is a binary variable equal to 1 if the respondent knows that respiratory droplets are a route of COVID-19 transmission.

[c] Close Contact Spread is a binary variable equal to 1 if the respondent knows that being within six feet of someone who is infected is a way to contract COVID-19.

[d] Asymptomatic Spread is a binary variable equal to 1 if the respondent knows whether COVID-19 can be transmitted by people without symptoms.

[e] Placebo Symptoms is a binary variable equal to 1 if the respondent endorsed any of the symptoms that are not frequently associated with COVID-19 (e.g. watery eyes) as one of the "top 3 symptoms".

[f] COVID-19 Hoax is a binary variable, which is equal to 1 if the respondent believes the virus is not real.

[g] Household Hospital Use is a binary variable, which is equal to 1 if the respondent or respondent’s family member has a health condition that requires frequent hospital use.

[h] Hotspot is a binary variable equal to 1 for New York City, New Orleans, Detroit, and Seattle.

[i] High health risk is a binary variable equal to 1 if the respondent endorses a diagnosis of chronic lung disease, cardiovascular disease or diabetes.

[j] Risk preference is a categorical variable from 0-10 which refers to a respondent’s preference for risk, in general.

© 2020 Alsan M et al. *JAMA Network Open.*
